# The role of birth weight on the causal pathway to child and adolescent ADHD symptomatology: a population‐based twin differences longitudinal design

**DOI:** 10.1111/jcpp.12949

**Published:** 2018-07-12

**Authors:** Kai Xiang Lim, Chao‐Yu Liu, Tabea Schoeler, Charlotte A.M. Cecil, Edward D. Barker, Essi Viding, Corina U. Greven, Jean‐Baptiste Pingault

**Affiliations:** ^1^ Social, Genetic, and Developmental Psychiatry Centre Institute of Psychiatry, Psychology and Neuroscience King's College London London UK; ^2^ Department of Clinical, Educational and Health Psychology Division of Psychology and Language Sciences University College London London UK; ^3^ School of Medicine National Taiwan University Taipei City Taiwan; ^4^ Department of Psychology Institute of Psychiatry, Psychology & Neuroscience King's College London London UK; ^5^ Department of Cognitive Neuroscience Donders Institute for Brain, Cognition and Behaviour Radboud University Medical Centre Nijmegen The Netherlands; ^6^ Karakter Child and Adolescent Psychiatry University Center Nijmegen The Netherlands

**Keywords:** Attention‐deficit/hyperactivity disorder, inattention, hyperactivity/impulsivity, birth weight, twin differences

## Abstract

**Background:**

Available evidence points towards lower birth weight as a risk factor for the development of attention deficit/hyperactivity disorder (ADHD) symptoms. We probed the causal nature of this putative effect of birth weight on ADHD symptoms using the twin differences design, which accounts for genetic and shared environmental confounds.

**Method:**

In a large population‐based twin sample – 3,499 monozygotic (MZ) and 6,698 dizygotic (DZ) pairs – parents, teachers or twins rated the twins’ ADHD symptoms at nine assessment waves (2–16 years). We implemented the twin differences design, which completely accounts for shared environmental and genetic confounding in MZ twins. We tested whether: (a) the lighter‐born twins had elevated ADHD symptoms compared to the heavier‐born twins, by regressing within‐pair differences of ADHD symptoms on within‐pair differences of birth weight among MZ twins; (b) the effect of birth weight on ADHD was moderated by gender, gestational age and low birth weight; (c) this effect changed with age at ADHD assessment using adapted latent growth curve models; and (d) results differed for inattention and hyperactivity/impulsivity.

**Results:**

Birth weight significantly predicted ADHD symptoms from early childhood to late adolescence. The lighter‐born twin had more ADHD symptoms than the heavier‐born cotwin among MZ twins across assessment waves and raters. No moderation effect was detected. The magnitude of the effect of birth weight decreased significantly across time for hyperactivity/impulsivity, but the decrease failed to reach significance for inattention. Estimates for inattention were significantly larger than for hyperactivity/impulsivity at each time point, implying stronger effect of birth weight on inattention symptoms.

**Conclusions:**

Our findings provide stringent evidence for environmental effect of lower birth weight on the causal pathway to elevated ADHD symptoms. Effect of birth weight persists across a 14‐year period from childhood into late adolescence, in particular for inattention symptoms.

## Introduction

Birth weight is an index of foetal growth and a marker of the prenatal environment (Schlotz & Phillips, 2009). Lower birth weight is associated with a range of adverse mental health and behavioural outcomes, such as lower intelligence quotient (IQ; e.g. Breslau, 1995), autistic‐like features (e.g. Ronald, Happé, Dworzynski, Bolton, & Plomin, 2010), conduct problems (e.g. Wiles et al., 2006) and attention‐deficit/hyperactivity disorder (ADHD; e.g. Schlotz, Jones, Godfrey, & Phillips, 2008). The adverse outcomes associated with lower birth weight can be viewed in light of the developmental origins of health and disease (DOHaD) hypothesis (Barker, 1997, 1998). It proposes that changes in prenatal environment during sensitive periods of organ development can result in long‐term structural or physiological alterations, which subsequently increase the risk of diseases such as psychiatric disorders later in life (Barker, 1997, 1998).

Numerous epidemiological studies have shown that lower birth weight is associated with ADHD symptoms (Botting, Powls, Cooke, & Marlow, 1997; Breslau et al., 1996; Mick, Biederman, Prince, Fischer, & Faraone, 2002; Nigg & Breslau, 2007). Despite this, the reported associations may not reflect a direct causal relationship as they might be confounded by genetic and shared environmental factors. For example, ADHD and birth weight are both heritable (76% and 40%, respectively; Clausson, Lichtenstein, & Cnattingius, 2000; Faraone et al., 2005). Although previous studies found no evidence of common genetic liability, residual genetic confounding of the association between birth weight and ADHD is still possible (Groen‐Blokhuis, Middeldorp, Van Beijsterveldt, & Boomsma, 2011; Pettersson et al., 2015). In addition, shared environmental factors associated with both birth weight and ADHD, such as socioeconomic status (Blumenshine, Egerter, Barclay, Cubbin, & Braveman, 2010; Foulon et al., 2015) and prenatal smoking (Galéra et al., 2011; Thapar et al., 2009) may also confound this association. To further probe the nature of the relationship between birth weight and ADHD symptoms, the twin differences design – a genetically informed design for causal inference – can be implemented (McGue, Osler, & Christensen, 2010; Pingault et al., 2018). This design capitalizes on the twins’ characteristics to control for shared environmental and genetic confounding, partially in dizygotic (DZ) twins and fully in monozygotic (MZ) twins. Findings from this design can provide stringent estimates of the role of birth weight on the causal pathway to developing ADHD symptoms.

A number of twin differences studies in childhood have demonstrated that lighter‐born twins have elevated ADHD symptoms compared to heavier‐born twins (Asbury, Dunn, & Plomin, 2006; Ficks, Lahey, & Waldman, 2013; Groen‐Blokhuis et al., 2011; Hultman et al., 2007; Lehn et al., 2007; Pettersson et al., 2015). However, these studies were limited in several key ways. First, no twin differences study used longitudinal methods to examine whether the effect of birth weight on ADHD symptoms persisted in the long‐term (i.e. from childhood to adolescence). The DOHaD hypothesis implies that this effect should persist in the long‐term (Barker, 1997, 1998). However, the effects of birth weight on adverse developmental outcomes may attenuate with age. For example, catch‐up growth in childhood and adolescence appears to alter the initial effects of lower birth weight on general cognitive and psychological performance in males (Lundgren, Cnattingius, Jonsson, & Tuvemo, 2001), although this is not observed for attention problems (Groen‐Blokhuis et al., 2011). To date, there is no longitudinal twin differences study examining whether the effect of birth weight persists from childhood to later stages of life.

A second key limitation is that no previous twin differences study formally tested whether birth weight may differentially influence the development of the two ADHD symptom dimensions, i.e. inattention and hyperactivity/impulsivity. A singleton study showed that adolescents with low birth weight had significantly higher inattention problems when compared to controls but no difference in hyperactivity was detected (Indredavik et al., 2004). This effect might persist until young adulthood, as another singleton study found that 20‐year‐old adults born with very low birth weight had more inattention problems but not hyperactivity problems compared to controls (Hack et al., 2004). In their twin differences study, Pettersson et al. (2015) suggested that lower birth weight affects both ADHD symptom dimensions in childhood, but whether the magnitude of this effect differs significantly across both dimensions remains untested. Also, most of the twin differences studies only used parents’ ratings to assess ADHD symptoms (Ficks et al., 2013; Groen‐Blokhuis et al., 2011; Hultman et al., 2007; Pettersson et al., 2015). This is potentially problematic as parental reports are prone to contrast effect, that is parents tend to highlight differences between twins (Thapar, Hervas, & McGuffin, 1995).

Lastly, a recent meta‐analysis systematically investigated moderators of the association between birth weight and ADHD (Momany, Kamradt, & Nikolas, 2018), which were not examined in previous twin differences studies. In the current study, we tested whether the size of this association differed according to gender, gestational age, and whether it differed for low birth weight (<2,500 g) versus normal birth weight participants. Although the meta‐analysis reported nonsignificant results for these three moderators, we aimed to confirm this finding within the twin differences design. This is the first large population‐based twin differences study to examine the differential effect of birth weight on both ADHD symptom dimensions, assessed by multiple informants across a 14‐year period. We tested whether the relationship between birth weight and ADHD symptoms:

1. remained after controlling for genetic and shared environmental confounds;
2. was moderated by gender, gestational age and low birth weight;
3. persisted from childhood to adolescence; and
4. whether differential effect of birth weight on the two ADHD symptom dimensions could be detected.

## Methods

### Participants

Participants were drawn from the Twins Early Development Study (TEDS), a large longitudinal study of twins born in England and Wales between 1994 and 1996, which is representative of the UK population as shown in Table S1 (Haworth, Davis, & Plomin, 2013). Their zygosity was determined using a parent‐rated instrument, which is 95% as accurate as DNA markers (Price et al., 2000). Analyses were conducted after excluding twin pairs with extreme perinatal conditions, severe medical conditions, uncertain zygosity, unknown gender, incomplete birth weight record and no data on ADHD symptoms (see footnote of Table S2 for details about exclusion criteria). The final sample included 10,197 twin pairs (6,698 DZ pairs, 3,499 MZ pairs, 51.2% females). The number of twin pairs included in each analysis ranged from 3,176 pairs to 7,119 pairs (see Table 1), depending on age, ADHD scale and informants. The present study included nine waves of assessments when twins were between 2 and 16 years old. Written informed consent was obtained from all participating families. This study was approved by the Institute of Psychiatry, King's College London, Ethics Committee.

**Table 1:** Phenotypic and MZ twin difference estimates of the relationship between birth weight and ADHD symptoms

Age	Scale	Phenotypic estimate, β (95% CI)	MZ estimate, β (95% CI)	Total *N* (MZ)
Parents’ ratings				
2	**BPBQ**	**−.086 (−.106, −.065)**	**−.126 (−.178, −.074)**	5,562 (1,910)
3	**BPBQ**	**−.078 (−.097, −.057)**	**−.190 (−.241, −.143)**	5,423 (1,876)
4	**BPBQ**	**−.073 (−.090, −.056)**	**−.193 (−.242, −.145)**	7,119 (2,445)
4	**SDQ**	**−.075 (−.091, −.058)**	**−.193 (−.245, −.139)**	7,113 (2,445)
7	**SDQ**	**−.066 (−.083, −.048)**	**−.237 (−.290, −.184)**	7,011 (2,524)
8	**CPRS total**	**−.052 (−.071, −.032)**	**−.115 (−.153, −.078)**	6,112 (2,177)
8	**CPRS H/I**	**−.049 (−.068, −.029)**	**−.064 (−.096, −.031)**	6,110 (2,177)
8	**CPRS IA**	**−.046 (−.065, −.026)**	**−.146 (−.190, −.101)**	6,109 (2,177)
9	**SDQ**	**−.074 (−.101, −.048)**	**−.169 (−.221, −.114)**	3,176 (1,176)
12	**SDQ**	**−.057 (−.077, −.036)**	**−.157 (−.203, −.114)**	5,458 (1,992)
12	**CPRS total**	**−.044 (−.063, −.023)**	**−.095 (−.133, −.056)**	5,463 (1,987)
12	**CPRS H/I**	**−.033 (−.053, −.013)**	**−.043 (−.074, −.012)**	5,461 (1,987)
12	**CPRS IA**	**−.045 (−.065, −.024)**	**−.122 (−.173, −.077)**	5,463 (1,986)
14	**CPRS total**	**−.030 (−.056, −.003)**	**−.090 (−.140, −.053)**	3,194 (1,232)
14	**CPRS H/I**	**−**.026 (**−**.051, .000)	**−.047 (−.084, −.010)**	3,189 (1,231)
14	**CPRS IA**	**−.027 (−.055, −.001)**	**−.108 (−.174, −.059)**	3,193 (1,232)
16	**SDQ**	**−**.024 (**−**.046, .000)	**−.112 (−.163, −.057)**	4,699 (1,705)
16	**CPRS total**	**−**.021 (**−**.044, .001)	**−.097 (−.141, −.050)**	4,706 (1,708)
16	**CPRS H/I**	**−.033 (−.056, −.011)**	**−.038 (−.082, −.001)**	4,704 (1,707)
16	**CPRS IA**	**−**.006 (**−**.029, .017)	**−.120 (−.173, −.065)**	4,705 (1,708)
Teachers’ ratings				
–	**Mean SDQ**	−.010 (−.028, .009)	**−.067 (−.107, −.028)**	7,049 (2,523)
Self‐report				
–	**Mean SDQ**	.016 (−.002, .034)	**−.121 (−.177, −.065)**	6,783 (2,429)

*N* = number of twin pairs for each analysis. H/I  =  Hyperactivity/impulsivity. IA = inattention. BPBQ = Behar's Preschool Behaviour Questionnaire. SDQ = Strength and Difficulties Questionnaire. CPRS‐R = Conners’ Parent Rating Scale ‐ Revised. Estimates in bold are significant. Teachers’ and self‐report ratings were obtained based on the average ratings across different ages. Note that the CI of the MZ estimate for CPRS‐R H/I (16 years) is very close to zero and hence this finding should be treated with caution.

### Measures

#### Birth weight

During first contact, parents reported each twin's birth weight in pounds or kilograms, which were then converted into grams for analyses. Twins’ median age was 1.6 years when birth weight data were collected.

#### ADHD symptoms

ADHD symptoms were assessed using age‐appropriate parent‐reported questionnaires, which are Behar's Preschool Behaviour Questionnaire (BPBQ; Behar & Stringfield, 1974) at ages 2, 3 and 4 years, the hyperactivity‐inattention subscale of Strength and Difficulties Questionnaire (SDQ; Goodman, 1997) at ages 4, 7, 9, 12 and 16 years, and the DSM‐IV‐based ADHD subscale of the Conners’ Parent Rating Scale – Revised (CPRS‐R; Conners, 2001) at ages 8, 12, 14 and 16 years. There were also teacher‐reported questionnaires (SDQ at ages 7, 9 and 12 years) and self‐reported questionnaires (SDQ at ages 9, 12 and 16 years). All scales are standard ADHD scales with adequate psychometric properties (see footnote of Table S3 for additional details on individual scales).

### Statistical analyses

All analyses were conducted using R Version 3.3.1 (R Core Team, 2016) and its Structural Equation Modelling package Lavaan Version 0.5‐22 (Rosseel, 2012).

#### Twin differences analyses

Using the twin differences design, we tested whether birth weight predicted ADHD symptoms at each wave of assessment. In order to examine the whole spectrum of birth weight differences, twin pairs were included irrespective of within‐twin pair differences in birth weight.

Response rates were systematically lower for teacher and self‐reports than for parental reports at the same data collection point. We therefore created two SDQ composite scores across ages to maximize sample size (i.e. averaging teacher‐rated SDQ measures at ages 7, 9 and 12 years and averaging self‐report SDQ measures at ages 9, 12 and 16 years). We did not include teacher and self‐reported scales available at only one data collection point. Prior to analysis for each ADHD scale, twins with missing ADHD data on that particular scale were excluded and all variables were standardized.

Two types of estimates were obtained: (a) the unadjusted phenotypic estimates from the whole twin sample and (b) the estimates from twin differences in MZ twins (MZ estimates). To obtain unadjusted phenotypic estimates, nonindependence of data within twin pairs were accounted for by allowing within‐twin pair correlations (Carlin, Gurrin, Sterne, Morley, & Dwyer, 2005). MZ estimates were obtained by conducting Ordinary Least Square (OLS) regressions through origin, regressing within‐twin pair differences in ADHD symptoms on within‐twin pair differences in birth weight (Carlin et al., 2005). MZ estimates are standardized beta coefficients from these regressions. The estimates from twin differences in DZ same‐gender twins were also obtained in a similar way for comparison (see Table S4).

To account for non‐normality and nonindependence, robust 95% confidence intervals (CI) were obtained using bootstrapping (10,000 repetitions) for all estimates. The moderating effects of gender, gestational age and low birth weight (<2,500 g) on these estimates were tested (see Tables S5–S7).

Unadjusted phenotypic estimates are bivariate correlations between birth weight and ADHD symptoms without controlling for any potential genetic and shared environmental confounds. MZ twins share 100% of their segregated genes and shared environment. Hence, MZ estimates are more robustly adjusted, accounting fully for both genetic and shared environmental influences.

#### Modelling developmental effect

Latent growth curve modelling was used to investigate if the change in MZ estimates was significant across development. In each MZ twin pair, one twin was assigned as ‘heavier twin’ and another as ‘lighter twin’. 171 MZ twin pairs with equal birth weight were excluded. The development of ADHD symptoms in lighter versus heavier twins was then modelled from age 8 to age 16 years, using the CPRS‐R, which provides a comprehensive assessment of both ADHD dimensions based on the DSM‐IV, and has been used extensively in ADHD research (Chang, Wang, & Tsai, 2016). Analyses were conducted for the total ADHD symptoms and repeated for the two symptom dimensions. The growth model included linear and quadratic components to account for nonlinear change. Missing data were handled using full information maximum likelihood (FIML) method.

To examine the developmental trend, we investigated whether:

1. the differences in ADHD symptoms between ‘lighter’ and ‘heavier’ twins were significant at age 8 and 16 years (i.e. difference between the mean in lighter twins vs. the mean in heavier twins, tested at each age); and
2. whether this difference in means between ‘lighter’ and ‘heavier’ twins was significantly larger at age 8 compared to age 16 years (difference observed at age 8 years minus difference at age 16 years). Computed estimates for (i) and (ii) were bootstrapped 10,000 times to obtain 95% CI.

#### Differential effect on symptom dimensions

To test whether the MZ estimates significantly differed depending on ADHD symptom dimensions, further analyses were carried out in MZ twins using the CPRS‐R, which has nine items for each symptom dimension. Two models were fitted for each of the symptom dimensions assessed at each age. In the first model, the MZ estimates for both symptom dimensions were constrained to be the same. The second model did not include equality constraints, thereby allowing both MZ estimates to be different. The difference in goodness of fit was then tested using the Satorra‐Bentler Chi‐square test. A significant difference would indicate that the effect of birth weight differs for the two dimensions.

## Results

### Twin differences analyses

Descriptive sample statistics regarding mean birth weight, number of twins and gender proportion for each ADHD scale are shown in Table S3. The mean gestational age was 36.47 weeks for the whole sample and 36.21 weeks for MZ twins.

As shown in Table 1, most phenotypic estimates were significant during childhood (e.g. at age 4 years, the estimate for parent‐rated SDQ was β* *= −.075, 95% CI: −.091, −.058), but became nonsignificant in adolescence (e.g. at age 16 years, the estimate for parent‐rated SDQ was β* *= −.024, 95% CI: −.046, .000). In contrast, despite being the most stringent type of estimates, all MZ estimates were significant from early childhood to adolescence with small effect sizes ranging from β* *= −.038 (95% CI: −.082, −.001) to β* *= −.237 (95% CI: −.290, −.184). These effect sizes were not moderated by gender, gestational age and low birth weight (see Tables S5–S7). Complementary analyses showed that a difference in 1 kg in birth weight corresponded to a difference of 0.42 symptom count for inattention and 0.17 symptom count for hyperactivity/impulsivity at age 8 years (see Table S8 for estimates at all ages). The effect of birth weight on ADHD was not affected by twins with very low birth weight (<1,500 g; see Table S9). Excluding the twins with extreme perinatal and severe medical conditions also did not change the direction and significance of the estimates (see Table S2).

### Modelling developmental effect

**Figure 1:**
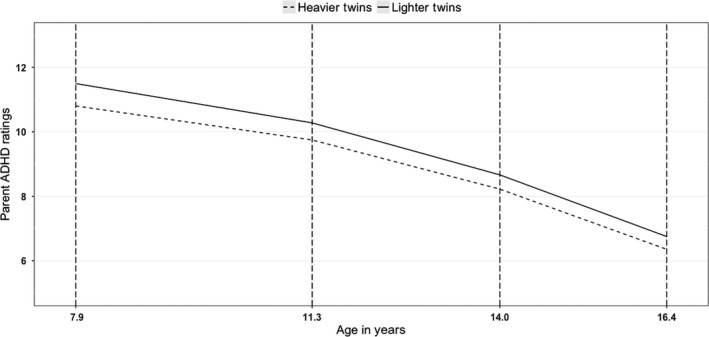
Predicted ADHD symptoms of MZ twins for CPRS‐R from age 8 to 16 years

The change in ADHD symptoms for heavier versus lighter‐born MZ twins is shown in Figure 1 for total ADHD symptoms (see Figure S1 for inattention and Figure S2 for hyperactivity/impulsivity).

At age 8 years, there was a significant difference of ADHD symptoms between heavier and lighter‐born twins. Lighter‐born twins tended to have higher total ADHD, inattention and hyperactivity/impulsivity symptoms (see Table 2). At age 16 years, lighter‐born twins tended to have higher total ADHD and inattention symptoms. Differences between heavier and lighter‐born twins were larger at age 8 years than 16, consistent with a lessening effect of birth weight over time. This decrease in effect sizes was observed for total and hyperactivity/impulsivity symptoms but failed to reach significance for inattention symptoms (see Table 2). The growth parameters are presented in Table S10.

**Table 2:** Latent growth curve modelling parameters for total ADHD, inattention and hyperactivity/impulsivity symptoms

	Predicted estimates (95% CI)		
	Heavier twins	Lighter twins	Within‐twin Difference of ADHD symptoms
Total ADHD symptoms			
Mean at age 8 years	10.80 (10.44, 11.18)	11.50 (11.11, 11.88)	**−.70 (−.91, −.48)**
Mean at age 16 years	6.36 (6.04, 6.70)	6.75 (6.43, 7.10)	**−.40 (−.62, −.15)**
Difference of means (8 vs. 16 years)	–	–	**−.30 (−.59, −.01)**
Inattention symptoms			
Mean at age 8 years	5.00 (4.81, 5.21)	5.44 (5.23, 5.65)	**−.44 (−.57, −.30)**
Mean at age 16 years	3.78 (3.57, 3.99)	4.09 (3.88, 4.31)	−**.31 (**−**.48,** −**.15)**
Difference of means (8 vs. 16 years)	–	–	−.12 (−.31, .07)
Hyperactivity/impulsivity symptoms			
Mean at age 8 years	5.79 (5.59, 6.00)	6.05 (5.84, 6.27)	**−.26 (−.36, −.15)**
Mean at age 16 years	2.57 (2.41, 2.73)	2.65 (2.49, 2.81)	−.08 (−.19, .03)
Difference of means (8 vs. 16 years)	–	–	**−.17 (−.32, −.04)**

Estimates in bold are significant. Heavier MZ twins had a mean birth weight of 2,592 g, whereas lighter MZ twins had a mean birth weight of 2,283 g.

### Differential effect on symptom dimensions

**Figure 2:**
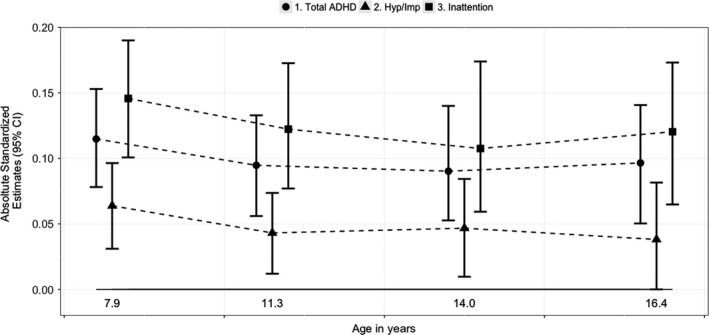
Absolute standardized MZ estimates with 95% CI for CPRS‐R total ADHD, hyperactivity/impulsivity (Hyp/Imp) and inattention symptoms across ages 8, 12, 14 and 16 years. All estimates are absolute values (negatively signed estimates are presented in Table 1). The larger the estimates, the greater the effect of birth weight on ADHD symptoms

Figure 2 shows that MZ estimates for inattention symptoms appear larger in magnitude than for hyperactivity/impulsivity. Formal Satorra‐Bentler tests revealed that the unconstrained models fit the data better than the constrained models from age 8 years to 16 years, suggesting that the stronger effect of birth weight on inattention compared to hyperactivity/impulsivity was significant and enduring (see Table 3).

**Table 3:** Satorra‐Bentler test results of the differential effect of birth weight on ADHD symptom dimensions

Age	Model	*df*	χ^2^	χ^2^ (*df*) difference	*p*‐value
8	Unconstrained	2	1.60	17.32 (1)	<.001***
	Constrained	3	24.14		
12	Unconstrained	2	.02	12.74 (1)	<.001***
	Constrained	3	14.90		
14	Unconstrained	2	2.40	4.44 (1)	.035*
	Constrained	3	7.84		
16	Unconstrained	2	.69	8.84 (1)	.003**
	Constrained	3	12.86		

*df*, degrees of freedom.

*p < .05; **p < .01; ***p < .001.

## Discussion

In the current twin differences study, we report four main findings. First, the epidemiological association observed between birth weight and ADHD symptomatology was confirmed in stringent twin differences analyses using MZ twins to control for genetic and shared environmental confounding. This points towards a plausible role of birth weight on the causal pathway leading to the development of ADHD symptoms. Second, this effect was not moderated by gender, gestational age and low birth weight. Third, the effect of birth weight on ADHD symptoms persisted from childhood to adolescence. Fourth, this effect was stronger and more persistent for inattention compared to hyperactivity/impulsivity.

The present findings corroborate previous twin studies as the effect of birth weight on ADHD symptoms remained significant even in MZ analyses, which control for all shared environmental and genetic confounding. Our findings add to the literature in that we used a large population‐based sample of twins, with consistent findings across multiple ADHD scales and multiple informants. In addition, we found that the effect of birth weight was not moderated by gender, gestational age and low birth weight, which confirms findings from a recent meta‐analysis (Momany et al., 2018). The small MZ estimates found in this study are the rule rather than exception in MZ differences analyses. Momany et al. (2018) reported a small overall correlation between birth weight and ADHD symptoms (*r* = −.15), with a correlation of *r* = −.09 when considering population‐based studies only. This may reflect that the environmental architecture underlying ADHD is just as complex as their genetic architecture, which involves small effects of many genetic variants (Demontis et al., 2017).

Intriguingly, in the current study MZ estimates were larger than phenotypic correlations between birth weight and ADHD symptoms. This pattern of findings replicates the results of a previous twin differences study (Pettersson et al., 2015), suggesting that our finding is not sample specific. Because the twin differences design stringently controls for genetic and shared environmental confounds, MZ twin differences estimates are typically lower than phenotypic estimates. Therefore, higher MZ estimates may suggest a suppression effect, i.e. confounders hide the true effect of birth weight as a risk factor for ADHD symptoms. Interestingly, the aforementioned meta‐analysis also reported a slightly larger effect size (*r *=* *−.18) in covariate‐adjusted analyses compared to unadjusted analyses (*r *=* *−.15, Momany et al., 2018). As the twin differences design accounts for unobserved confounders, the level of adjustment is more stringent than in classical epidemiological designs. It may thus not be surprising that the differences between phenotypic and MZ estimates appear larger in our study than the difference between adjusted and unadjusted estimates in the meta‐analysis.

Longitudinal findings in the current study add to current knowledge in that the effect of lower birth weight on ADHD symptoms were persistent from age 2 to age 16 years, in particular for inattention symptoms. These findings partially support the DoHAD hypothesis (Barker, 1997, 1998), stipulating that prenatal environment constitutes a long‐term health risk. Different mechanisms may explain our findings. For example, prenatal ischemia hypoxia (i.e. insufficient nutrients and oxygen supply in utero) is a primary pathway to lower birth weight, and it also produces lasting changes in neurodevelopmental functioning, which increases risk for ADHD (Smith, Schmidt‐Kastner, McGeary, Kaczorowski, & Knopik, 2016). In addition to showing persisting effect of birth weight in adolescence for total ADHD and inattention symptoms, we also revealed ‘catch‐up’ or developmental compensation effects, whereby early effect of birth weight decrease over time (significantly for total ADHD and hyperactivity/impulsivity symptoms).

The stronger phenotypic association between birth weight and inattention than hyperactivity/impulsivity has been reported in previous studies (Hack et al., 2004; Indredavik et al., 2004) and was confirmed here using the twin differences design. We found that the effect of birth weight on inattention symptoms persisted into adolescence, whereas the effect on hyperactivity/impulsivity symptoms decreased, which further supported the differential effect of birth weight on the two symptom dimensions.

### Limitations

Our findings point towards the importance of birth weight –a marker for foetal growth– in the aetiology of long‐term ADHD symptoms. However, birth weight is a complex risk factor, and further research is needed to identify which aspects of foetal growth might explain the findings (e.g. by identifying restricted development in specific brain areas). In addition, other prenatal unmeasured nonshared environmental factors associated with foetal growth, such as positioning in the womb, placentation, or differences in nutritional availability could also explain the findings (Plomin, DeFries, Knopik, & Neiderhiser, 2013). Nonshared differences within pairs after birth may be a direct consequence of within‐twin pair differences in birth weight (e.g. additional medical care for the lighter twin). Such postnatal differences may therefore lie on indirect paths from birth weight to ADHD symptoms, either buffering or accentuating the effect of birth weight, which warrants further investigation. Another limitation is that the current study included retrospective parental reports of birth weight, which may be affected by recall bias. However, retrospective parental reports of birth weight have been shown to be reliable up to 30 years postbirth (Catov et al., 2006; Lumey, Stein, & Ravelli, 1994).

## Conclusion

Restricted foetal development, as indexed by lower birth weight, may play a role in the causal pathways leading to the development of ADHD symptomatology. Whereas this effect was found to decrease across time for total ADHD and hyperactivity/impulsivity symptoms, the decrease failed to reach significance for inattention symptoms. In addition, birth weight was found to influence inattention symptoms more strongly than hyperactivity/impulsivity symptoms across childhood and adolescence.

**Key points**

- Using a longitudinal twin differences design, we probed the causal nature of the observed association between birth weight and ADHD symptoms.
- Lower birth weight predicted higher ADHD symptoms over a 14‐year period from childhood to adolescence.
- The size of the effect of birth weight decreased significantly for total ADHD and hyperactivity/impulsivity symptoms.
- A stronger and more persistent effect of birth weight was found for inattention symptoms compared to hyperactivity/impulsivity symptoms.
- Additional research to further dissect the mechanisms explaining this environmentally driven relationship between birth weight and ADHD symptoms are required.

## Supporting information


**Table S1.** Sample characteristics.
**Table S2.** Phenotypic, DZ and MZ twin difference estimates without excluding twins with exclusion criteria.
**Table S3.** Descriptive statistics for birth weight and each of the ADHD scales.
**Table S4.** Phenotypic, DZ and MZ twin difference estimates of the relationship between birth weight and ADHD symptoms.
**Table S5.** Moderating effect of gender.
**Table S6.** Moderating effect of short gestational age (<37 weeks).
**Table S7.** Moderating effect of low birth weight (<2,500 g).
**Table S8.** Corresponding change in symptoms for change in 1 kg.
**Table S9.** Phenotypic, DZ and MZ twin difference estimates after excluding twins with very low birth weight (<1,500 g).
**Table S10.** Descriptive statistics for parameters in latent growth curve modelling.
**Figure S1.** Predicted inattention levels of monozygotic twins for Conners’ Parent Rating Scale‐Revised from age 8 to 16 years.
**Figure S2.** Predicted hyperactivity/impulsivity levels of monozygotic twins for Conners’ Parent Rating Scale‐Revised from age 8 to 16 years.
