# Supplementary material for: The role of birth weight on the causal pathway to child and adolescent ADHD symptomatology: a population‐based twin differences longitudinal design
Source: J Child Psychol Psychiatry. 2018 Jul 12;59(10):1036–43. doi: 10.1111/jcpp.12949 (PMC6175300; doi:10.1111/jcpp.12949)
Supplement: Supplementary file 1 — Table S1. Sample characteristics. Table S2. Phenotypic, DZ and MZ twin difference estimates without excluding twins with exclusion criteria. Table S3. Descriptive statistics for birth weight and each of the ADHD scales. Table S4. Phenotypic, DZ and MZ twin difference estimates of the relationship between birth weight and ADHD symptoms. Table S5. Moderating effect of gender. Table S6. Moderating effect of short gestational age (<37 weeks). Table S7. Moderating effect of low birth weight (<2,500 g). Table S8. Corresponding change in symptoms for change in 1 kg. Table S9. Phenotypic, DZ and MZ twin difference estimates after excluding twins with very low birth weight (<1,500 g). Table S10. Descriptive statistics for parameters in latent growth curve modelling. Figure S1. Predicted inattention levels of monozygotic twins for Conners’ Parent Rating Scale‐Revised from age 8 to 16 years. Figure S2. Predicted hyperactivity/impulsivity levels of monozygotic twins for Conners’ Parent Rating Scale‐Revised from age 8 to 16 years. [file JCPP-59-1036-s001.pdf]

Supplementary Material

Table S1. Sample Characteristics.

Table S2. Phenotypic, DZ and MZ twin difference estimates without excluding twins with exclusion criteria.

Table S3. Descriptive statistics for birth weight and each of the ADHD scales.

Table S4. Phenotypic, DZ and MZ twin difference estimates of the relationship between birth weight and ADHD symptoms.

Table S5. Moderating effect of sex.

Table S6. Moderating effect of short gestational age (< 37 weeks).

Table S7. Moderating effect of low birth weight (<2,500 grams).

Table S8. Corresponding change in symptoms for change in one kilogram.

Table S9. Phenotypic, DZ and MZ twin difference estimates after excluding twins with very low birth weight (<1,500 grams).

Table S10. Descriptive statistics for parameters in latent growth curve modelling.

Figure S1. Predicted inattention levels of monozygotic twins for Conners' Parent Rating Scale-Revised from age 8 years to 16 years.

Figure S2. Predicted hyperactivity/impulsivity levels of monozygotic twins for Conners' Parent Rating Scale-Revised from age 8 years to 16 years.

Table S1.

Sample Characteristics.

	Returned data (N families)	% White	% Mothers with A-levels or higher	% Mother employed	% Father employed	% Female	% MZ
UK census	-	93%	32%	49%	89%	-	34.3%
TEDS first contact	13,722	91.7%	35.5%	43.1%	91.6%	50.1%	33.2%
TEDS study sample	10,201	92.9%	37.7%	44.3%	92.4%	51.2%	34.3%

Note. UK data from the 2000 General Household Survey (Kovas, Haworth, Dale, & Plomin, 2007; ONS, 2001) are used rather than more recent data because they provide more appropriate comparisons for TEDS twins who were born from year 1994 to 1996. The % MZ data are from Imaizumi (2003) because they are not available from the UK census data. A-levels are the national educational exam taken at 18 years of age in the UK. MZ = monozygotic twins.

Table S2.

Phenotypic, DZ and MZ twin difference estimates without excluding twins with exclusion criteria.

Age	Scale	Phenotypic estimate, β (95% CI)	DZ estimate, β (95% CI)	MZ estimate, β (95% CI)	Total N (DZSS, MZ)
Parents' ratings					
2	**BPBQ**	**-.080 (-.099, -.060)**	**-.188 (-.263, -.114)**	**-.141 (-.194, -.087)**	5920 (1975, 1999)
3	**BPBQ**	**-.081 (-.101, -.062)**	**-.182 (-.268, -.099)**	**-.207 (-.259, -.157)**	5760 (1911, 1962)
4	**BPBQ**	**-.082 (-.098, -.065)**	**-.197 (-.273, -.123)**	**-.209 (-.257, -.159)**	7602 (2552, 2565)
4	**SDQ**	**-.080 (-.097, -.064)**	**-.195 (-.273, -.115)**	**-.203 (-.256, -.149)**	7596 (2548, 2565)
7	**SDQ**	**-.074 (-.091, -.056)**	**-.186 (-.264, -.109)**	**-.259 (-.313, -.206)**	7457 (2442, 2640)
8	**CPRS total**	**-.068 (-.087, -.049)**	**-.108 (-.167, -.051)**	**-.116 (-.153, -.076)**	6453 (2100, 2275)
8	**CPRS H/I**	**-.060 (-.080, -.041)**	**-.079 (-.139, -.020)**	**-.061 (-.094, -.025)**	6451 (2100, 2275)
8	**CPRS IA**	**-.065 (-.084, -.045)**	**-.118 (-.187, -.050)**	**-.151 (-.195, -.107)**	6450 (2099, 2275)
9	**SDQ**	**-.082 (-.108, -.056)**	**-.195 (-.289, -.103)**	**-.178 (-.232, -.125)**	3293 (1065, 1208)
12	**SDQ**	**-.060 (-.080, -.039)**	**-.159 (-.237, -.087)**	**-.177 (-.226, -.132)**	5667 (1839, 2050)
12	**CPRS total**	**-.049 (-.069, -.028)**	**-.133 (-.191, -.077)**	**-.112 (-.153, -.073)**	5672 (1845, 2045)
12	**CPRS H/I**	**-.036 (-.056, -.016)**	**-.093 (-.154, -.031)**	**-.055 (-.088, -.022)**	5670 (1844, 2045)
12	**CPRS IA**	**-.050 (-.070, -.030)**	**-.146 (-.211, -.083)**	**-.143 (-.193, -.095)**	5672 (1844, 2044)
14	**CPRS total**	**-.035 (-.061, -.009)**	**-.137 (-.218, -.058)**	**-.098 (-.146, -.058)**	3286 (1062, 1261)
14	**CPRS H/I**	**-.030 (-.055, -.005)**	**-.105 (-.198, -.017)**	**-.055 (-.094, -.016)**	3281 (1060, 1260)
14	**CPRS IA**	**-.033 (-.060, -.006)**	**-.136 (-.217, -.056)**	**-.113 (-.177, -.064)**	3285 (1061, 1261)
16	**SDQ**	**-.025 (-.048, -.003)**	**-.123 (-.206, -.039)**	**-.126 (-.182, -.070)**	4936 (1591, 1769)
16	**CPRS total**	**-.025 (-.048, -.003)**	**-.084 (-.153, -.013)**	**-.111 (-.160, -.063)**	4943 (1592, 1772)
16	**CPRS H/I**	**-.036 (-.058, -.014)**	-.035 (-.110, .036)	**-.054 (-.099, -.014)**	4941 (1592, 1771)
16	**CPRS**	-.011	**-.104**	**-.132**	4942

	**IA**	(-.034, .012)	**(-.181, -.024)**	**(-.189, -.071)**	(1591, 1772)
Teachers' rating					
-	**Mean SDQ**	**-.024 (-.042, -.006)**	**-.066 (-.129, -.004)**	**-.074 (-.116, -.035)**	7428 (2454, 2633)
Self-report					
-	**Mean SDQ**	.014 (-.004, .031)	**-.109 (-.176, -.042)**	**-.134 (-.191, -.078)**	7129 (2324, 2520)

Note. N = number of twins for each analysis. DZSS = DZ same-sex twins. H/I = Hyperactivity/impulsivity. IA= inattention. BPBQ = Behar's Preschool Behaviour Questionnaire. SDQ = Strength and Difficulties Questionnaire. CPRS-R = Conners' Parent Rating Scale - Revised. Estimates in bold are significant. Teachers' and self-report ratings were obtained based on the average ratings across different ages. This analysis includes 689 twin pairs who were excluded from all other analyses. Among these 689 twin pairs, 484 twin pairs were excluded due to severe medical conditions [(1) autism/ASD; (2) cerebral palsy; (3) any genetic, chromosomal or inherited disorder; (4) brain damage or disorders affecting brain function; (5) Downs syndrome; (6) profound deafness; (7) global developmental delay; (8) complete blindness; (9) death of either twin), 169 twin pairs due to having perinatal outlier [(1) low birth weight (< 471grams); (2) short gestational age (< 27 weeks); (3) maternal drinking during pregnancy (>= 14units/week); (4) long period of special care after birth (> 97 days); (5) long stay in hospital after birth (> 74 days)], and 36 twin pairs due to both reasons.

Table S3.

Descriptive statistics for birth weight and each of the ADHD scales.

Age	Scale		N	Mean	SD	Skew	Kurtosis
**0**	**Birth weight**	Whole sample	12,173	2497.51	533.39	-0.22	3.08
		Same-sex DZ sample	4,003	2522.84	526.34	-0.23	3.09
		MZ sample	4,104	2433.06	535.87	-0.16	2.99
**Parents' ratings**							
**2**	**BPBQ**	Whole sample	5,562	2.94	1.97	0.34	2.46
		Same-sex DZ sample	1,836	3.00	2.04	0.36	2.39
		MZ sample	1,910	2.96	1.88	0.29	2.51
**3**	**BPBQ**	Whole sample	5,423	2.79	1.97	0.43	2.51
		Same-sex DZ sample	1,785	2.85	2.03	0.45	2.48
		MZ sample	1,876	2.79	1.88	0.35	2.51
**4**	**BPBQ**	Whole sample	7,119	2.60	1.93	0.58	2.74
		Same-sex DZ sample	2,372	2.66	1.99	0.56	2.62
		MZ sample	2,445	2.61	1.84	0.62	2.95
**4**	**SDQ**	Whole sample	7,113	3.96	2.31	0.43	2.69
		Same-sex DZ sample	2,368	3.99	2.41	0.42	2.57
		MZ sample	2,445	4.04	2.17	0.44	2.89
**7**	**SDQ**	Whole sample	7,011	3.56	2.53	0.58	2.61
		Same-sex DZ sample	2,285	3.61	2.57	0.52	2.49
		MZ sample	2,524	3.55	2.46	0.61	2.73
**8**	**CPRS-R total**	Whole sample	6,112	10.92	9.07	1.40	5.22
		Same-sex DZ sample	1,977	10.99	9.03	1.32	4.95
		MZ sample	2,177	10.96	8.94	1.40	5.22

**8**	**CPRS-R H/I**	Whole sample	6,110	5.68	4.99	1.34	4.86
		Same-sex DZ sample	1,977	5.67	5.02	1.29	4.62
		MZ sample	2,177	5.83	4.97	1.32	4.79
**8**	**CPRS-R IA**	Whole sample	6,109	5.25	4.99	1.46	5.28
		Same-sex DZ sample	1,976	5.32	4.99	1.37	4.98
		MZ sample	2,177	5.13	4.82	1.46	5.37
**9**	**SDQ**	Whole sample	3,176	3.22	2.36	0.83	3.20
		Same-sex DZ sample	1,023	3.19	2.37	0.82	3.17
		MZ sample	1,176	3.26	2.29	0.85	3.31
**12**	**SDQ**	Whole sample	5,458	2.80	2.25	0.87	3.41
		Same-sex DZ sample	1,765	2.85	2.30	0.83	3.26
		MZ sample	1,992	2.76	2.16	0.88	3.52
**12**	**CPRS-R total**	Whole sample	5,463	9.70	8.43	1.59	6.26
		Same-sex DZ sample	1,771	9.85	8.63	1.63	6.39
		MZ sample	1,987	9.57	8.23	1.53	5.93
**12**	**CPRS-R-H/I**	Whole sample	5,461	4.22	4.30	1.73	6.77
		Same-sex DZ sample	1,770	4.31	4.44	1.73	6.66
		MZ sample	1,987	4.20	4.23	1.65	6.37
**12**	**CPRS-R IA**	Whole sample	5,463	5.48	5.05	1.37	5.05
		Same-sex DZ sample	1,770	5.54	5.14	1.40	5.16
		MZ sample	1,986	5.37	4.85	1.30	4.82
**14**	**CPRS-R total**	Whole sample	3,194	8.46	8.14	1.69	6.70
		Same-sex DZ sample	1,033	8.66	8.14	1.66	6.76
		MZ sample	1,232	8.00	7.52	1.60	6.49
**14**	**CPRS-R H/I**	Whole sample	3,189	3.46	4.01	1.96	7.92

		Same-sex DZ sample	1,031	3.51	4.04	1.98	8.12
		MZ sample	1,231	3.30	3.72	1.82	7.26
**14**	**CPRS-R IA**	Whole sample	3,193	4.99	5.05	1.46	5.30
		Same-sex DZ sample	1,032	5.15	5.07	1.37	5.04
		MZ sample	1,232	4.70	4.68	1.44	5.55
**16**	**SDQ**	Whole sample	4,699	2.23	1.96	1.18	4.51
		Same-sex DZ sample	1,517	2.29	2.05	1.13	4.22
		MZ sample	1,705	2.11	1.83	1.24	4.87
**16**	**CPRS-R total**	Whole sample	4,706	6.67	7.30	2.03	8.51
		Same-sex DZ sample	1,517	7.01	7.62	1.98	7.94
		MZ sample	1,708	6.03	6.72	1.97	8.24
**16**	**CPRS-R H/I**	Whole sample	4,704	2.56	3.44	2.43	10.83
		Same-sex DZ sample	1,517	2.69	3.63	2.38	10.23
		MZ sample	1,707	2.40	3.20	2.26	9.56
**16**	**CPRS-R IA**	Whole sample	4,705	4.11	4.79	1.77	6.66
		Same-sex DZ sample	1,516	4.32	4.98	1.71	6.26
		MZ sample	1,708	3.63	4.36	1.89	7.51
**Teachers' ratings**							
**-**	**Mean SDQ**	Whole sample	7,049	2.64	2.38	1.11	3.65
		Same-sex DZ sample	2,320	2.68	2.41	1.07	3.53
		MZ sample	2,523	2.61	2.32	1.14	3.80
**Self-report**							
**-**	**Mean SDQ**	Whole sample	6,783	3.65	2.02	0.45	2.87
		Same-sex DZ sample	2,202	3.71	2.05	0.43	2.77
		MZ sample	2,429	3.52	1.95	0.49	3.00

Note. H/I = Hyperactivity/impulsivity. IA= inattention. BPBQ = Behar's Preschool Behaviour Questionnaire. SDQ = Strength and Difficulties Questionnaire. CPRS-R =

Conners’ Parent Rating Scale - Revised. Teachers’ and self-report ratings were obtained based on the average ratings across different ages. Birth weight sample is the number of twins with complete birth weight record prior to the exclusion of twins without complete ADHD data, and hence the birth weight sample size is larger than total number of twins (N = 10,197). The BPBQ consists of four items, two pertaining to hyperactivity/impulsivity (e.g. “Squirmy, fidgety”) and two pertaining to inattention (e.g. “Has poor concentration, or short attention span”). Twins were rated by their parents on a three-point Likert scale ranging from (0) “not true” to (2) “certainly true”. The SDQ contains five items, three for hyperactivity/impulsivity (e.g. “Restless, overactive, cannot stay still for long”) and two for inattention (e.g. “Easily distracted, concentration wanders”). All items were rated on a three-point Likert scale, ranging from (0) “not true” to (2) “certainly true”. The CPRS-R includes nine items for hyperactivity-impulsivity (e.g. “leaves seat in classroom or in other situations in which remaining seated is expected”) and another nine for inattention (e.g. “has difficulties sustaining attention in tasks or activities”). Each item on CPRS-R was rated on a 4-point Likert scale, ranging from (0) “Not true at all” to (3) “Very much true”.

Table S4.

Phenotypic, DZ and MZ twin difference estimates of the relationship between birth weight and ADHD symptoms.

Age	Scale	Phenotypic estimate, β (95% CI)	DZ estimate, β (95% CI)	MZ estimate, β (95% CI)	Total N (DZSS, MZ)
Parents' ratings					
2	**BPBQ**	**-.086 (-.106, -.065)**	**-.189 (-.265, -.116)**	**-.126 (-.178, -.074)**	5,562 (1,836, 1,910)
3	**BPBQ**	**-.078 (-.097, -.057)**	**-.147 (-.231, -.065)**	**-.190 (-.241, -.143)**	5,423 (1,785, 1,876)
4	**BPBQ**	**-.073 (-.090, -.056)**	**-.164 (-.237, -.090)**	**-.193 (-.242, -.145)**	7,119 (2,372, 2,445)
4	**SDQ**	**-.075 (-.091, -.058)**	**-.162 (-.241, -.083)**	**-.193 (-.245, -.139)**	7,113 (2,368, 2,445)
7	**SDQ**	**-.066 (-.083, -.048)**	**-.143 (-.218, -.071)**	**-.237 (-.290, -.184)**	7,011 (2,285, 2,524)
8	**CPRS total**	**-.052 (-.071, -.032)**	**-.076 (-.132, -.021)**	**-.115 (-.153, -.078)**	6,112 (1,977, 2,177)
8	**CPRS H/I**	**-.049 (-.068, -.029)**	-.053 (-.113, .005)	**-.064 (-.096, -.031)**	6,110 (1,977, 2,177)
8	**CPRS IA**	**-.046 (-.065, -.026)**	**-.086 (-.151, -.023)**	**-.146 (-.190, -.101)**	6,109 (1,976, 2,177)
9	**SDQ**	**-.074 (-.101, -.048)**	**-.165 (-.260, -.074)**	**-.169 (-.221, -.114)**	3,176 (1,023, 1,176)
12	**SDQ**	**-.057 (-.077, -.036)**	**-.126 (-.199, -.056)**	**-.157 (-.203, -.114)**	5,458 (1,765, 1,992)
12	**CPRS total**	**-.044 (-.063, -.023)**	**-.104 (-.161, -.047)**	**-.095 (-.133, -.056)**	5,463 (1,771,1,987)
12	**CPRS H/I**	**-.033 (-.053, -.013)**	**-.068 (-.130, -.007)**	**-.043 (-.074, -.012)**	5,461 (1,770, 1,987)
12	**CPRS IA**	**-.045 (-.065, -.024)**	**-.118 (-.180, -.053)**	**-.122 (-.173, -.077)**	5,463 (1,770, 1,986)

14	**CPRS total**	**-.030 (-.056, -.003)**	**-.123 (-.204, -.044)**	**-.090 (-.140, -.053)**	3,194 (1,033, 1,232)
14	**CPRS H/I**	-.026 (-.051, .000)	-.086 (-.176, .000)	**-.047 (-.084, -.010)**	3,189 (1,031, 1,231)
14	**CPRS IA**	**-.027 (-.055, -.001)**	**-.129 (-.211, -.049)**	**-.108 (-.174, -.059)**	3,193 (1,032, 1,232)
16	**SDQ**	-.024 (-.046, .000)	**-.121 (-.204, -.040)**	**-.112 (-.163, -.057)**	4,699 (1,517, 1,705)
16	**CPRS total**	-.021 (-.044, .001)	**-.073 (-.141, -.003)**	**-.097 (-.141, -.050)**	4,706 (1,517, 1,708)
16	**CPRS H/I**	**-.033 (-.056, -.011)**	-.040 (-.111, .034)	**-.038 (-.082, -.001)**	4,704 (1,517, 1,707)
16	**CPRS IA**	-.006 (-.029, .017)	**-.083 (-.158, -.006)**	**-.120 (-.173, -.065)**	4,705 (1,516, 1,708)
Teachers' rating					
-	**Mean SDQ**	-.010 (-.028, .009)	-.045 (-.109, .017)	**-.070 (-.107, -.028)**	7,049 (2,320, 2,523)
Self-report					
-	**Mean SDQ**	.0163 (-.002, .034)	**-.086 (-.154, -.020)**	**-.121 (-.177, -.065)**	6,783 (2,202, 2,429)

Note. N = number of twins for each analysis. DZSS = DZ same-sex twins. H/I = Hyperactivity/impulsivity. IA= inattention. BPBQ = Behar's Preschool Behaviour Questionnaire. SDQ = Strength and Difficulties Questionnaire. CPRS-R = Conners' Parent Rating Scale - Revised. Estimates in bold are significant. Teachers' and self-report ratings were obtained based on the average ratings across different ages

Table S5.
Moderating effect of sex.

		Male Model			Female Model			Model comparison		
Age	Scale	*β*	95% CI		*β*	95% CI		$\Delta\chi^2$	*p*	*p* adjusted
2	BPBQ	-.099	-.172	-.027	-.149	-.222	-.077	.910	.340	.859
3	BPBQ	-.216	-.294	-.138	-.168	-.231	-.105	.885	.347	.859
4	BPBQ	-.162	-.234	-.089	-.221	-.285	-.157	1.445	.229	.859
4	SDQ	-.160	-.241	-.079	-.224	-.292	-.156	1.405	.236	.859
7	SDQ	-.220	-.300	-.140	-.253	-.325	-.181	.364	.547	.859
8	CPRS-Total	-.122	-.185	-.058	-.109	-.151	-.067	.105	.746	.959
8	CPRS H/I	-.061	-.113	-.009	-.066	-.106	-.027	.026	.871	.959
8	CPRS IA	-.161	-.236	-.086	-.133	-.184	-.082	.365	.546	.859
9	SDQ	-.204	-.288	-.119	-.138	-.204	-.071	1.435	.231	.859
12	SDQ	-.157	-.228	-.086	-.157	-.214	-.099	.000	.996	.996
12	CPRS-Total	-.075	-.138	-.013	-.111	-.158	-.065	.811	.368	.859
12	CPRS H/I	-.028	-.079	.023	-.056	-.093	-.019	.733	.392	.859
12	CPRS IA	-.103	-.179	-.028	-.139	-.199	-.078	.513	.474	.859
14	CPRS-Total	-.080	-.140	-.021	-.098	-.158	-.038	.163	.686	.943
14	CPRS H/I	-.037	-.099	.026	-.054	-.098	-.010	.203	.653	.943
14	CPRS IA	-.100	-.174	-.026	-.114	-.194	-.033	.061	.805	.959
16	SDQ	-.118	-.210	-.026	-.107	-.170	-.044	.034	.853	.959
16	CPRS-Total	-.095	-.174	-.016	-.098	-.150	-.045	.002	.961	.996
16	CPRS H/I	-.064	-.130	.002	-.019	-.069	.031	1.119	.290	.859
16	CPRS IA	-.100	-.194	-.005	-.136	-.196	-.075	.399	.527	.859
-	Mean SDQ (t)	-.084	-.148	-.020	-.051	-.097	-.005	.666	.415	.859
-	Mean SDQ (c)	-.078	-.165	.009	-.159	-.230	-.089	2.030	.154	.859

Note. $\Delta\chi^2$ = Chi-squared difference. MZ = monozygotic. H/I = Hyperactivity/impulsivity. IA= inattention. BPBQ = Behar's Preschool Behaviour Questionnaire. SDQ = Strength and Difficulties Questionnaire. CPRS-R = Conners'

Parent Rating Scale - Revised. Teachers' (t) and self-report (c) ratings were obtained based on the average ratings across different ages. Generalised estimating equation (GEE) estimates were obtained by modelling estimates of within-twin pair and between-twin pair effect (Carlin, Gurrin, Sterne, Morley, & Dwyer, 2005). The within-twin pair estimates are reported in this table, with 95% CI obtained from bootstrapping (10,000 times). The within-twin pair estimates in male and female models are equivalent to the male and female MZ twin difference estimates, which correspond to the effect of birth weight on ADHD symptoms. Model comparisons were conducted by using Wald-Chi-squared test, comparing the male/female models with and without interaction terms between sex and within-twin pair effect.

Table S6

Moderating effect of short gestational age (< 37 weeks).

		Long GA Model			Short GA Model			Model comparison		
Age	Scale	β	95% CI		β	95% CI		$\Delta\chi^2$	p	p adjusted
2	BPBQ	-.134	-.203	-.065	-.113	-.190	-.037	.154	.695	.999
3	BPBQ	-.138	-.202	-.073	-.253	-.329	-.177	5.149	.023	.513
4	BPBQ	-.150	-.214	-.086	-.245	-.317	-.173	3.750	.053	.581
4	SDQ	-.180	-.251	-.109	-.214	-.291	-.137	.406	.524	.999
7	SDQ	-.219	-.292	-.146	-.254	-.333	-.175	.409	.522	.999
8	CPRS-Total	-.128	-.174	-.082	-.099	-.158	-.041	.555	.456	.999
8	CPRS H/I	-.076	-.116	-.036	-.050	-.100	.001	.636	.425	.999
8	CPRS IA	-.157	-.213	-.102	-.132	-.202	-.061	.319	.572	.999
9	SDQ	-.178	-.245	-.110	-.156	-.240	-.072	.159	.690	.999
12	SDQ	-.149	-.202	-.095	-.165	-.240	-.090	.123	.726	.999
12	CPRS-Total	-.098	-.143	-.052	-.090	-.154	-.027	.037	.848	.999
12	CPRS H/I	-.043	-.081	-.005	-.043	-.093	.007	.000	.999	.999
12	CPRS IA	-.128	-.184	-.072	-.114	-.195	-.034	.075	.784	.999
14	CPRS-Total	-.105	-.153	-.057	-.071	-.147	.006	.554	.457	.999
14	CPRS H/I	-.063	-.109	-.016	-.025	-.084	.035	.976	.323	.999
14	CPRS IA	-.118	-.181	-.056	-.094	-.194	.007	.168	.682	.999
16	SDQ	-.118	-.179	-.057	-.100	-.191	-.008	.109	.741	.999
16	CPRS-Total	-.094	-.141	-.046	-.098	-.180	-.015	.007	.932	.999
16	CPRS H/I	-.037	-.081	.007	-.041	-.112	.031	.008	.930	.999

16	CPRS IA	-.117	-.176	-.058	-.120	-.215	-.026	.003	.957	.999
-	Mean SDQ (t)	-.050	-.099	-.001	-.086	-.147	-.024	.782	.376	.999
-	Mean SDQ (c)	-.138	-.210	-.067	-.103	-.190	-.016	.379	.538	.999

Note. $\Delta\chi^2$ = chi-squared difference. GA=gestational age. MZ = monozygotic. H/I = Hyperactivity/impulsivity. IA= inattention. BPBQ = Behar's Preschool Behaviour Questionnaire. SDQ = Strength and Difficulties Questionnaire. CPRS-R = Conners' Parent Rating Scale - Revised. Teachers' (t) and self-report (c) ratings were obtained based on the average ratings across different ages. Generalised estimating equation (GEE) estimates were obtained by modelling estimates of within-twin pair and between-twin pair effect (Carlin, Gurrin, Sterne, Morley, & Dwyer, 2005). The within-twin pair estimates are reported in this table, with 95% CI obtained from bootstrapping (10,000 times). The within-twin pair estimates in short GA (< 37 weeks) and long GA (≥ 37 weeks) models are equivalent to the MZ twin difference estimates for twin pairs with short and long GA respectively, which correspond to the effect of birth weight on ADHD symptoms. Model comparisons were conducted by using Wald-Chi-squared test, comparing the short/long GA models with and without interaction terms between GA and within-twin pair effect.

Table S7.

Moderating effect of low birth weight (<2,500 grams).

		High BW Model			Low BW Model			Model comparison		
Age	Scale	*β*	95% CI		*β*	95% CI		$\Delta\chi^2$	*p*	*P* adjusted
2	BPBQ	-.115	-.207	-.023	-.130	-.191	-.069	.070	.792	.909
**3**	**BPBQ**	**-.057**	**-.152**	**.038**	**-.232**	**-.289**	**-.174**	**9.477**	**.002**	**.046**
4	BPBQ	-.129	-.218	-.041	-.211	-.267	-.155	2.302	.129	.284
4	SDQ	-.144	-.245	-.044	-.208	-.269	-.147	1.112	.292	.492
7	SDQ	-.119	-.222	-.017	-.273	-.335	-.210	6.282	.012	.067
8	CPRS-Total	-.082	-.153	-.011	-.124	-.167	-.082	1.016	.313	.492
8	CPRS H/I	-.052	-.114	.009	-.067	-.104	-.030	.161	.688	.890
8	CPRS IA	-.098	-.177	-.018	-.160	-.212	-.108	1.633	.201	.403
9	SDQ	-.167	-.277	-.057	-.169	-.230	-.109	.002	.965	.981
12	SDQ	-.050	-.131	.031	-.186	-.239	-.134	7.631	.006	.063
12	CPRS-Total	-.028	-.092	.036	-.113	-.159	-.068	4.547	.033	.145
12	CPRS H/I	.007	-.049	.062	-.057	-.093	-.021	3.521	.061	.174
12	CPRS IA	-.053	-.133	.027	-.142	-.199	-.085	3.137	.077	.187
14	CPRS-Total	-.097	-.164	-.031	-.088	-.140	-.036	.048	.826	.909
14	CPRS H/I	-.075	-.157	.006	-.038	-.079	.003	.650	.420	.616
14	CPRS IA	-.098	-.174	-.021	-.111	-.180	-.041	.063	.801	.909
16	SDQ	-.011	-.089	.067	-.141	-.205	-.076	6.329	.012	.067
16	CPRS-Total	-.032	-.101	.038	-.115	-.170	-.061	3.449	.063	.174
16	CPRS H/I	-.018	-.094	.059	-.044	-.091	.003	.334	.563	.775
16	CPRS IA	-.036	-.121	.050	-.145	-.209	-.081	4.006	.045	.166
-	Mean SDQ (t)	-.068	-.145	.010	-.067	-.111	-.022	.001	.981	.981
-	Mean SDQ (c)	-.067	-.170	.035	-.137	-.202	-.072	1.249	.264	.483

Note. $\Delta\chi^2$ = chi-squared difference. BW=birth weight. MZ = monozygotic. H/I = Hyperactivity/impulsivity. IA= inattention. BPBQ = Behar's Preschool Behaviour

Questionnaire. SDQ = Strength and Difficulties Questionnaire. CPRS-R = Conners' Parent Rating Scale - Revised. Teachers' (t) and self-report (c) ratings were obtained based on the average ratings across different ages. Estimates in bold are significant at $p < .05$ significance level. Generalised estimating equation (GEE) estimates were obtained by modelling estimates of within-twin pair and between-twin pair effect (Carlin, Gurrin, Sterne, Morley, & Dwyer, 2005). The within-twin pair estimates are reported in this table, with 95% CI obtained from bootstrapping (10,000 times). The within-twin pair estimates in low BW (≤

2,500 grams) and high BW (> 2,500 grams) models are equivalent to the MZ twin difference estimates for twin pairs with low and high BW respectively, which correspond to the effect of birth weight on ADHD symptoms. Model comparisons were conducted by using Wald-Chi-squared test, comparing the low/high BW models with and without interaction terms between BW and within-twin pair effect.

Table S8.

Corresponding change in symptoms for change in one kilogram.

Symptoms	Age	Phenotypic estimate (95% CI)	DZ Estimate (95% CI)	MZ estimate (95% CI)
Inattention	8	-0.123 (-0.195,-0.054)	-0.305 (-0.557,-0.067)	-0.421 (-0.601,-0.245)
	12	-0.108 (-0.181,-0.030)	-0.359 (-0.611,-0.125)	-0.306 (-0.521,-0.101)
	14	-0.067 (-0.162,0.027)	-0.414 (-0.740,-0.121)	-0.332 (-0.571,-0.134)
	16	-0.026 (-0.095,0.042)	-0.136 (-0.370,0.106)	-0.215 (-0.401,-0.020)
Hyperactivity/ impulsivity	8	-0.173 (-0.242,-0.102)	-0.190 (-0.422,0.033)	-0.172 (-0.306,-0.039)
	12	-0.076 (-0.137,-0.014)	-0.150 (-0.361,0.062)	-0.167 (-0.296,-0.039)
	14	-0.052 (-0.123,0.015)	-0.276 (-0.548,-0.028)	-0.097 (-0.234,0.036)
	16	-0.064 (-0.111,-0.016)	-0.041 (-0.222,0.128)	-0.108 (-0.223,-0.008)

Note. The estimates illustrate the difference in number of symptoms within each twin pair when there is a one kilogram difference in birth weight.

Table S9.

Phenotypic, DZ and MZ twin difference estimates after excluding twins with very low birth weight (<1,500 grams).

Age	Scale	Phenotypic estimate, β (95% CI)	DZ estimate, β (95% CI)	MZ estimate, β (95% CI)	Total N (DZSS, MZ)
Parents' ratings					
2	**BPBQ**	-.075 (-.095,-.054)	-.155 (-.226,-.084)	-.102 (-.147,-.057)	5268 (1746,1776)
3	**BPBQ**	-.070 (-.090,-.050)	-.129 (-.205,-.053)	-.166 (-.212,-.120)	5150 (1708,1745)
4	**BPBQ**	-.061 (-.079,-.043)	-.137 (-.204,-.070)	-.164 (-.207,-.121)	6699 (2254,2266)
4	**SDQ**	-.061 (-.078,-.043)	-.131 (-.202,-.059)	-.168 (-.215,-.122)	6693 (2250,2266)
7	**SDQ**	-.057 (-.074,-.039)	-.117 (-.187,-.048)	-.200 (-.245,-.155)	6585 (2165,2340)
8	**CPRS total**	-.043 (-.062,-.024)	-.063 (-.117,-.008)	-.100 (-.129,-.071)	5751 (1889,2006)
8	**CPRS H/I**	-.038 (-.057,-.020)	-.047 (-.101,.007)	-.059 (-.085,-.032)	5750 (1889,2006)
8	**CPRS IA**	-.040 (-.060,-.021)	-.066 (-.128,-.005)	-.124 (-.159,-.090)	5748 (1888,2006)
9	**SDQ**	-.066 (-.092,-.039)	-.135 (-.228,-.042)	-.145 (-.195,-.095)	3000 (976,1092)
12	**SDQ**	-.044 (-.065,-.024)	-.090 (-.159,-.022)	-.130 (-.168,-.091)	5147 (1683,1846)
12	**CPRS total**	-.035 (-.055,-.015)	-.072 (-.128,-.015)	-.083 (-.114,-.052)	5150 (1688,1842)
12	**CPRS H/I**	-.026 (-.045,-.006)	-.040 (-.097,.017)	-.038 (-.065,-.010)	5148 (1687,1842)
12	**CPRS IA**	-.038 (-.058,-.017)	-.087 (-.150,-.024)	-.107 (-.145,-.070)	5150 (1687,1841)

14	**CPRS total**	-.035 (-.062,-.009)	-.091 (-.172,-.011)	-.084 (-.121,-.047)	3022 (990,1147)
14	**CPRS H/I**	-.032 (-.058,-.006)	-.054 (-.140,.032)	-.042 (-.077,-.006)	3018 (989,1146)
14	**CPRS IA**	-.033 (-.059,-.006)	-.103 (-.186,-.021)	-.101 (-.146,-.057)	3021 (989,1147)
16	**SDQ**	-.019 (-.041,.004)	-.089 (-.165,-.013)	-.105 (-.146,-.064)	4443 (1451,1586)
16	**CPRS total**	-.016 (-.038,.005)	-.043 (-.108,.022)	-.091 (-.126,-.055)	4447 (1450,1588)
16	**CPRS H/I**	-.024 (-.046,-.003)	-.014 (-.081,.053)	-.033 (-.069,.003)	4445 (1450,1587)
16	**CPRS IA**	-.006 (-.028,.016)	-.056 (-.126,.014)	-.115 (-.154,-.075)	4446 (1449,1588)
Teachers' rating					
-	**Mean SDQ**	.001 (-.017,.020)	-.020 (-.074,.035)	-.048 (-.085,-.012)	6623 (2213,2328)
Self-report					
-	**Mean SDQ**	.012 (-.007,.030)	-.067 (-.132,-.003)	-.115 (-.164,-.065)	6388 (2093,2250)

Note. Twin pairs with either twin having very low birth weight (< 1,500 grams) were excluded from this analysis. N = number of twins for each analysis. DZSS = DZ same-sex twins. H/I = Hyperactivity/impulsivity. IA= inattention. BPBQ = Behar's Preschool Behaviour Questionnaire. SDQ = Strength and Difficulties Questionnaire. CPRS-R = Conners' Parent Rating Scale - Revised. Estimates in bold are significant.Teachers' and self-report ratings were obtained based on the average ratings across different ages.

Table S10.

Descriptive statistics for parameters in latent growth curve modelling.

CPRS-R	Heavier twins			Lighter Twins		
	Intercept at age 8 years (95% CI)	Linear slope (95% CI)	Quadratic slope (95% CI)	Intercept at age 8 years (95% CI)	Linear slope (95% CI)	Quadratic slope (95% CI)
Total ADHD	10.80 (10.44, 11.18)	-.17 (-.29, -.04)	-.04 (-.06, -.03)	11.50 (11.11, 11.88)	-.23 (-.36, -.09)	-.04 (-.05, -.02)
I/A	5.00 (4.81, 5.21)	.28 (.20, .35)	-.05 (-.06, -.04)	5.44 (5.23, 5.65)	.23 (.15, .31)	-.05 (-.06, -.04)
H/I	5.79 (5.59, 6.00)	-.44 (-.51, -.37)	.01 (.00, .01)	6.05 (5.84, 6.27)	-.46 (-.53, -.39)	.01 (-.00, .01)

Note. The 95% CI were obtained through 10,000 bootstrapping.

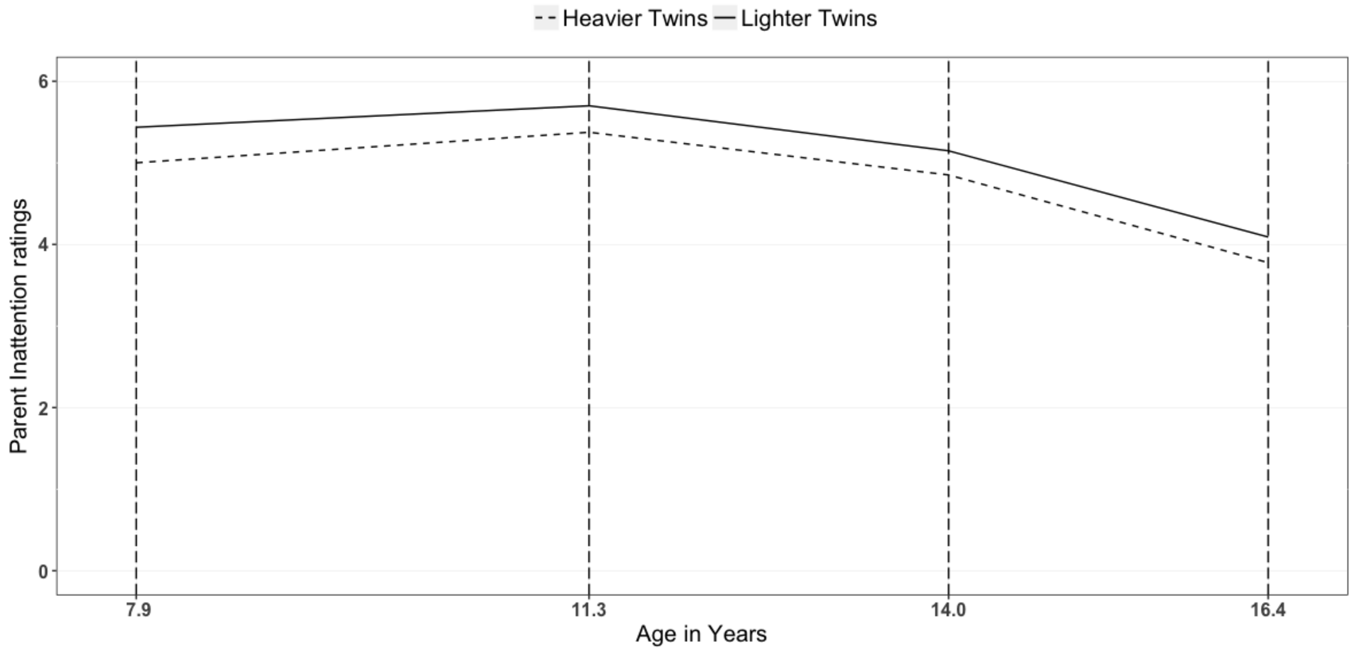

Figure S1: Predicted inattention levels of monozygotic twins for Conners' Parent Rating Scale-Revised from age 8 years to 16 years.

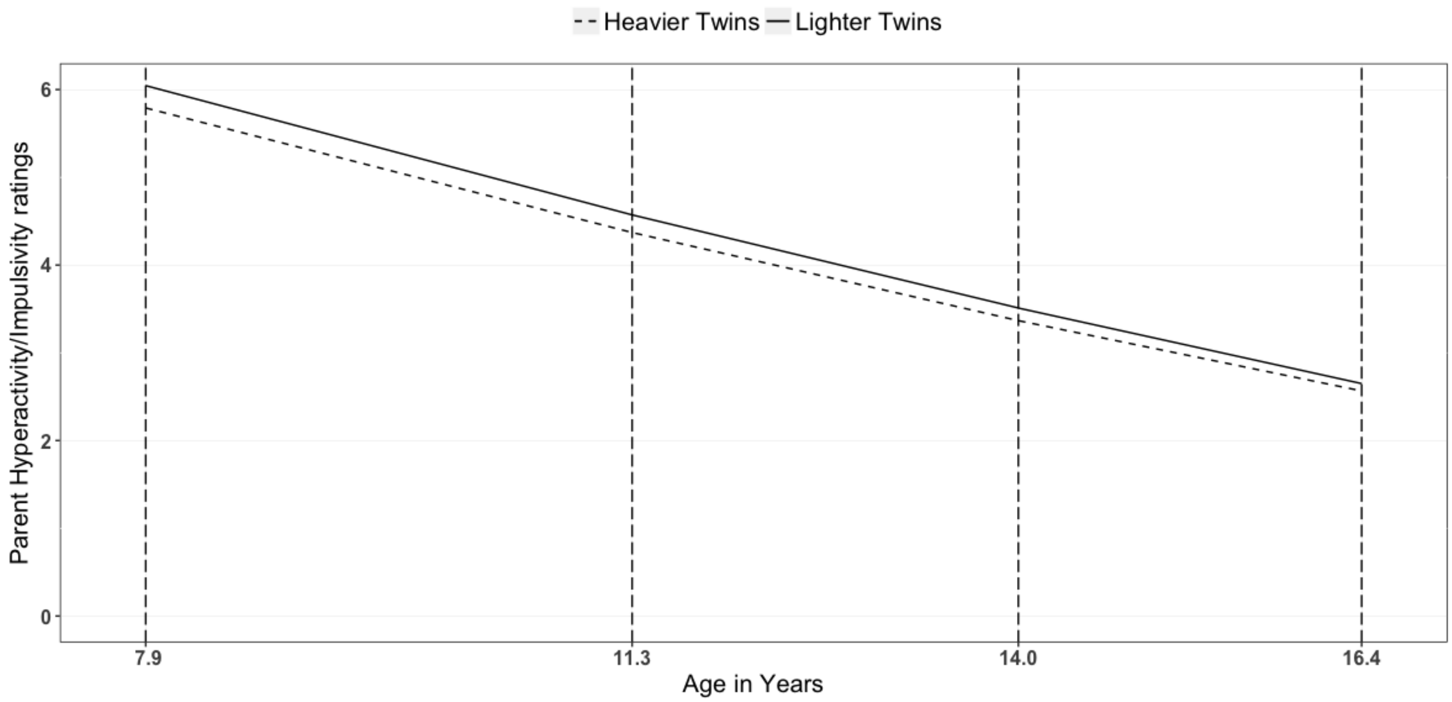

Figure S2: Predicted hyperactivity/impulsivity levels of monozygotic twins for Conners' Parent Rating Scale-Revised from age 8 years to 16 years.

References

Carlin, J. B., Gurrin, L. C., Sterne, J. A. C., Morley, R., & Dwyer, T. (2005). Regression models for twin studies: A critical review. *International Journal of Epidemiology*, *34*(5), 1089–1099. https://doi.org/10.1093/ije/dyi153

Imaizumi, Y. (2003). A comparative study of zygotic twinning and triplet rates in eight countries, 1972–1999. *Journal of Biosocial Science*, *35*(2), 287–302.

Kovas, Y., Haworth, C. M., Dale, P. S., & Plomin, R. (2007). The genetic and environmental origins of learning abilities and disabilities in the early school years. *Monographs of the Society for Research in Child Development*, *72*(3), vii-1.

ONS. (2001). *Living In Britain: Results from the 2000 General Household Survey*. London: Stationery Office.

Kovas, Y., Haworth, C. M., Dale, P. S., & Plomin, R. (2007). The genetic and environmental origins of learning abilities and disabilities in the early school years. *Monographs of the Society for Research in Child Development*, *72*(3), vii-1.

ONS. (2001). *Living In Britain: Results from the 2000 General Household Survey*. London: Stationery Office.
